# Child art psychotherapy in CAMHS: Which cases are referred and which cases drop out?

**DOI:** 10.1186/s40064-016-3509-2

**Published:** 2016-10-19

**Authors:** Leslie Saba, Alison Byrne, Aisling Mulligan

**Affiliations:** 1 Department of Child and Adolescent Psychiatry, University College Dublin, Belfield, Dublin 4, Ireland; 2 Department of Child Art Psychotherapy, Mater Miserircordiae University Hospital, Dublin 7, Ireland; 3 HSE Dublin North City and County Child and Adolescent Mental Health Service, James Joyce St., Dublin 1, Ireland

**Keywords:** Child art psychotherapy, Psychiatry, Child, Adolescent, Psychotherapeutic modality

## Abstract

**Background:**

The Vasarhelyi method of child art psychotherapy (CAP) is offered at certain Child and Adolescent Mental Health Services. Children attend three introductory sessions, and then choose to continue weekly CAP or conclude the sessions.

**Aims:**

This study aims to identify the clinical disorders and characteristics of patients referred to CAP, and to determine who engages with the therapy.

**Methods:**

A retrospective review of the clinical records of 67 children who attended CAP in DNCC/Mater CAMHS over 13 years was performed. The data was analysed using Microsoft Excel 12.0 and SPSS version 20.

**Results:**

67 children (57 % male and 43 % female) aged 5–17 years participated in CAP with an average age of 10.6 years. Children attended an average of 14 sessions of CAP, with a range of 1–61 sessions (mean of 13.8 ± 12.9 sessions). Anxiety disorder (28 %), behaviour disorder/ODD (25 %), and ADHD (21 %) are the most common diagnoses referred. These diagnoses along with autism spectrum disorder (ASD) had the highest overall engagement, while those with depression engaged the least. Children with ADHD and with ASD attended high numbers of sessions (with a mean of 23 and 19 respectively). Those who experienced acute life events or difficulties in the home engaged well (60 and 40 % respectively). There was no significant difference found in the percentage of appointments attended by males in comparison to females.

**Conclusion:**

CAP is generally acceptable to children, with a high average attendance rate. It was noted that children with ADHD and with ASD engaged well with the therapy for prolonged periods, whereas children with depression did not engage so well. We suggest that CAMHS clinics should consider referring children diagnosed with ADHD and children diagnosed with ASD to CAP as an adjunct to other therapies. We suggest that individuals with depression should be referred initially to other therapeutic services as the engagement with CAP was relatively poor.

## Background

The Vasarhelyi method of child art psychotherapy (CAP), created by Vera Vasarhelyi in 1982, is a psychotherapy modality which uses art to act as a bridge between the child’s unconscious thoughts and their hidden relationship with the image. The method has theoretical underpinnings in the writings of Freud (1891) and of Jung (1966, 1968) and is described in more detail in The Vasarhelyi Method of CAP in CAMHS: a survey of clinical supervisors by McGovern et al. (2016). The important features of the Vasarhelyi Method of CAP are as follows:

- Images are used for pictorial communication as a non-verbal language. It is acknowledged that children may find it easier to express their thoughts and emotions through the use of drawings. The young person is encouraged to express his/her feelings through artwork and to develop his/her own formulation of his/her feelings. The therapist facilitates the young person to develop his/her own formulation and does not externally impose on the young person.
- *An “Empty Space” is provided both physically and emotionally*. This helps the child to create the formulation of his/her own pictorial material without the conscious or unconscious influence of the therapist. The therapist allocates a time to sit aside or outside the room as the child creates an image. The therapist then joins the child; the child is asked to describe the image and discuss his or her feelings towards it.
- *The time aspect of pictorial thinking* differs from real time. Vasarhelyi outlines how verbal descriptions have a beginning, middle and end whereas one image can simultaneously show different time frames: “…however complex their contents might be they don’t need time to start, progress and finish … they transcend time in the sense that time is only involved in the contemplation of them; the picture itself is non-temporal,” (Vasarhelyi 1981). Vasarhelyi proposes that this facilitates easier access to the unconscious (Vasarhelyi 1990).
- *Three semi-structured assessment sessions* are offered where the child is asked to draw an image of him/herself, an image of his/her family, and an image of his/her earliest childhood memory. These sessions are sometimes the key to understanding the child’s current conflicts. The therapist then meets with the young person and his/her parent(s) to negotiate either to cease or to continue therapy until the child and therapist agree that it is no longer needed—the child has some autonomy in the decision about whether or not to continue therapy.

The Vasarhelyi method of therapy is client led, and it is important that a period of separation from the therapist occurs, during which the young person creates an image. The therapist then joins the young person and encourages him/her to interpret his/her own image. While the initial three assessment sessions are task orientated, all subsequent sessions are without specific tasks and the child is encouraged to create his/her images in whatever way he or she likes. The creation of images may be a form of free association for children, thus helping the child to access his/her unconscious. Like psychodynamic psychotherapy, the method has no suggested endpoint for therapy—the therapist and child will agree to an ending. The Vasarhelyi method of art psychotherapy draws on the theories of Jung, who described the power of the image to help access the unconscious and was considered by some to be the first art therapist (Jung 1966). The Vasarhelyi method is based on the principle that visual thinking has a unique and direct relationship with the unconscious: “Child art psychotherapy can facilitate a unique insight into the dynamics of the unconscious, and allow the privilege of seeing hidden processes, which would otherwise remain largely inaccessible to exploration” (Vasarhelyi 1990). The Vasarhelyi method also draws on the theories of Freud in his description of the unconscious (Freud 1891) and on the theories of Arlow, who recommended investigation of the connections between current mental experience and past events via investigation of the unconscious (Arlow 1995). As with other therapeutic modalities, provision of a safe space, clear boundaries and maintenance of confidentiality are essential components.

Interest in art therapy commenced in the care of traumatised adults and it should be noted that there is limited research on the effectiveness of art therapy in general. There is some evidence that art therapy may be an effective treatment for adults who have experienced trauma (Schouten et al. 2015) but there is limited evidence as to the effectiveness of art therapy in children who have experienced trauma (Wethington et al. 2008). However a review of the use of art therapy in children concluded that art therapy is generally accepted as an effective treatment for children who have experienced psychological distress (Reynolds et al. 2000).

Our CAMHS service is in an area of high social need, and a high non-attendance rate in the service is a challenge, with a non-attendance rate of 31 % noted in 2011 (FIOS Committee 2011), and a current non-attendance rate of approximately 20 %. There are various reported non-attendance rates at CAMHS services, with a non-attendance rate of 13 % reported in one CAMHS service in Ireland (Skokauskas et al. 2009) and non-attendance rates of 13–50 % reported for initial clinic appointments in other CAMHS services internationally (Skokauskas et al. 2009; Cottrell et al. 1998; Aubrey et al. 2003; Gould et al. 1985; Hoare et al. 1996; Brian and Cameron 2004). Attrition rates in CAMHS are also of interest, and attendance rates at the second offered appointment are sometimes lower than the attendance at the first offered appointment (Aubrey et al. 2003; Sodipo et al. 2008). Specific therapy services can have difficulties with enrolment and with engagement of client groups (Butler and Titus 2015) which may be an important issue when funding a service. We could find no study on the engagement of patients attending art therapy in children but we note that a systematic review of attendance at culturally adapted parent training for disruptive behaviour (Butler and Titus 2015) found 7 studies which reported attendance rates, which varied from 36 % (Gross et al. 2009) to 85 % (Coard et al. 2004). A recent study comparing cognitive-behavioral therapy (CBT), panic-focused psychodynamic psychotherapy (PFPP), and applied relaxation training (ART) for panic disorder with and without agoraphobia in adults reported attrition rates of 41 % for applied relaxation training, 25 % for CBT and 22 % for PFPP (Milrod et al. 2016). The high attrition rates in specific therapy methods may limit their overall effectiveness or cost effectiveness in CAMHS.

Therapists are trained in the Vasarhelyi method of CAP by attending a 2 year university based training programme with an 18 month clinical attachment to a Child and Adolescent Mental Health Service (CAMHS) team. Neither the child nor the therapist require previous training in art. The Vasarhelyi method of CAP has been in use in some CAMHS in Ireland for 14 years. While it is generally accepted that Art Therapy is a useful modality in children (Vasarhelyi 1990), we wished perform an evaluation of the use of the Vasarhelyi method of CAP in one CAMHS clinic in Ireland. We aim to (1) identify the clinical disorders and characteristics of patients who were referred to CAP, and (2) to determine if children engaged well with CAP. We describe a retrospective chart and clinic database review of the use of the Vasarhelyi method in one CAMHS in Ireland.

## Methods

### Study sample

The sample consisted of 77 children who were referred to CAP in Dublin North City and County/Mater CAMHS over the past 13 years and who had completed their attendance at CAP. 10 children were excluded from the final dataset as they attended no CAP sessions. Children were referred predominantly from two CAMHS teams, Team A and Team C, who are based in one city centre building.

### Procedure

Ethical approval was obtained before the commencement of this study from the Health Service Executive North City Ethics Committee, relevant to the CAMHS area. A list of children who attended CAP in the service was generated using the clinic database records. A list of relevant diagnosis of interest was generated by the third author with a plan that the children referred to CAP would be categorised according to those who had (1) depressive disorder, (2) anxiety disorder, (3) behavioural disorder/oppositional defiant disorder, (4) eating disorder, (5) ADHD/hyperkinetic disorder, (6) autism spectrum disorder (ASD), (7) no major mental illness (Table 1). A list of clinical characteristics of interest was generated, which could be categorised as present or absent in those who attended CAP: (a) deliberate self harm, (b) suicidal thinking, (c) attachment difficulties (d) emerging emotionally unstable personality disorder, (e) history of child sexual abuse, (f) out of home placement, (g) learning difficulties, (h) psychosocial environment difficulties (Table 1). A data collection form was generated to allow collection of demographic data (age and gender), the list of diagnostic categories, the list of clinical characteristics and data about the number of appointments attended and the number missed.

**Table 1:** Categorisation of psychiatric diagnoses and patient characteristics

Disorders	Characteristics
Depression	Deliberate self harm
Anxiety disorder	Suicidal thinking
Behavioural disorder/ODD	Attachment difficulties
Eating disorder	Child sexual abuse
ADHD	Out of home placement
Autism spectrum disorder	Learning difficulties
Emerging emotionally unstable personality disorder	Psychosocial environment
No major mental illness	History of child experiencing parental separation/distress

The clinic database system was used to retrieve the information specified on the data collection form regarding diagnoses, demographics and appointment data for all 77 children who completed attendance at CAP in the service. The integrity of the data was checked and verified by a senior psychiatrist (A.M.). The children’s charts were reviewed to verify and supplement information available from the clinic database. Data were collected, compared, and statistically analysed using Microsoft Office Excel 12.0 and using SPSS version 20.

### Data collection

#### Demographics

Demographic information was collected including the gender of the child, therapist identification, the child’s year of birth, and year of CAP commencement. The approximate age a child began CAP was calculated by subtracting the year of birth from the year of CAP commencement. Family status information was collected from the clinic database system and from chart review and was divided into those with “no parental separation or distress,” “with parental distress,” and “parental separation.” When information for a certain category is not present, an “unknown” option was selected.

#### Psychiatric diagnoses

Psychiatric diagnoses according to the International Classification of Mental and Behavioural Disorders 10 (ICD-10) diagnostic criteria (World Health Organization 1992) were retrieved from the clinic database system, where they had been inputted by the clinical treating team, and from the children’s charts. The information was re-categorised to the summary diagnostic items listed in the Data Collection Form as follows: (1) Individuals listed under “Mood (affective) disorders” were classified under depression, after a chart review to check that this was the appropriate diagnostic category. (2) Anyone with either of “Emotional disorders,” “Selective Mutism,” “Obsessive Compulsive Disorder,” “Nonorganic sleep disorders,” or “Post traumatic stress disorder” were listed under “anxiety disorder.” (3) Children with “Conduct disorders” or “Oppositional defiant disorder” were reclassified as “behaviour disorder/ODD.” (5) Those with “Hyperkinetic Disorders” were listed as ADHD/hyperkinetic disorder. (6) Those with “Pervasive developmental disorder,” “Autism,” or “Aspergers” were collectively classified as ASD. (7) Individuals who were listed as “No psychiatric illness” were classified as no major mental illness.

#### Clinical characteristics

Information on those who experienced (a) deliberate self harm, (b) suicidal thinking, (c) attachment difficulties, and (e) child sexual abuse (CSA) was obtained from chart reviews or clinician discussion and a yes/no option was selected. A clinician determined from chart review whether a child had (d) possible emerging emotionally unstable personality disorder. Children who were not living with their parents or in either of their father or mother’s house were classified as (f) “out of home placement.” Of the children classified as “out of home placement,” one was adopted at 4 years of age, and one was in the care of foster parents.

The category (g) learning difficulties was subdivided into subcategories as follows: those who had mild learning difficulty, those who had specific learning difficulties, those who had mild learning and specific learning difficulties, and those who had no learning needs. Those listed on the clinic database as ICD “Mild Mental Retardation” were categorised as mild learning difficulty; those on the clinic database who had “ICD—Specific developmental disorders of scholastic skills” were categorised as specific learning difficulties and those who had a combination of both were listed as mild and specific learning difficulties. If an individual has a normal IQ and “No specific disorder of psychological development,” they were said to have no learning needs.

Psychosocial environmental difficulties data were taken from ICD Axis V on the clinic database, which were re-categorised into four groups: Familial/Relationships/communication difficulties (in home), acute life events, societal stressors/stress school work (outside home), and those that had no significant distortion of the psychosocial environment. The ICD categories “Mental disorder/deviance of parent,” “Abnormal qualities of upbringing,” and “Stressful events/situations from child’s disability” were grouped into familial relationships/communication difficulties (in home). Other ICD categories that remained unchanged were acute life events, societal stressors/stress school work (outside home), and those that had no significant distortion of the psychosocial environment.

#### Appointment attendance

The total number of CAP appointments offered and the total number attended was recorded, using information from the clinic database for each child. Engagement was classified according to two measures depending on the total number of sessions attended and the attendance rate. Attendance rate was calculated by dividing the number of sessions attended by the total number of sessions offered. Those who attended 3 sessions or less, or had an attendance rate of 40 % or less were categorised as “did not engage.” Anyone who attended more than 3 sessions with an attendance rate between 40 and 70 % was categorised as “partial engagement.” Those who attended more than 3 sessions and had an attendance rate of 70 % or greater were categorised as “engaged with therapy” (Table 2).

**Table 2:** Classification of engagement with CAP

	Did not engage	Partial engagement	Engaged
Number of sessions	≤3	>3	>3

	OR	AND	AND
Attendance rate (%)	≤40	>40 but <70	≥70

### Data analysis

The data were analysed according to (1) diagnoses, (2) clinical characteristics, and (3) attendance at CAMHS. The average number of sessions attended was calculated.

## Results

### Demographics

67 children (57 % male and 43 % female) aged 5–17 years participated in CAP with an average age of 10.6 years. Children attended an average of 14 sessions of CAP, with a range of 1–61 sessions (mean of 13.8 ± 12.9 sessions). There were 16 therapists in total. No significant effect of age (in years) on the percentage of appointments attended was found, using a one way analysis of variance.

### Psychiatric diagnoses and clinical characteristics

**Fig. 1:**
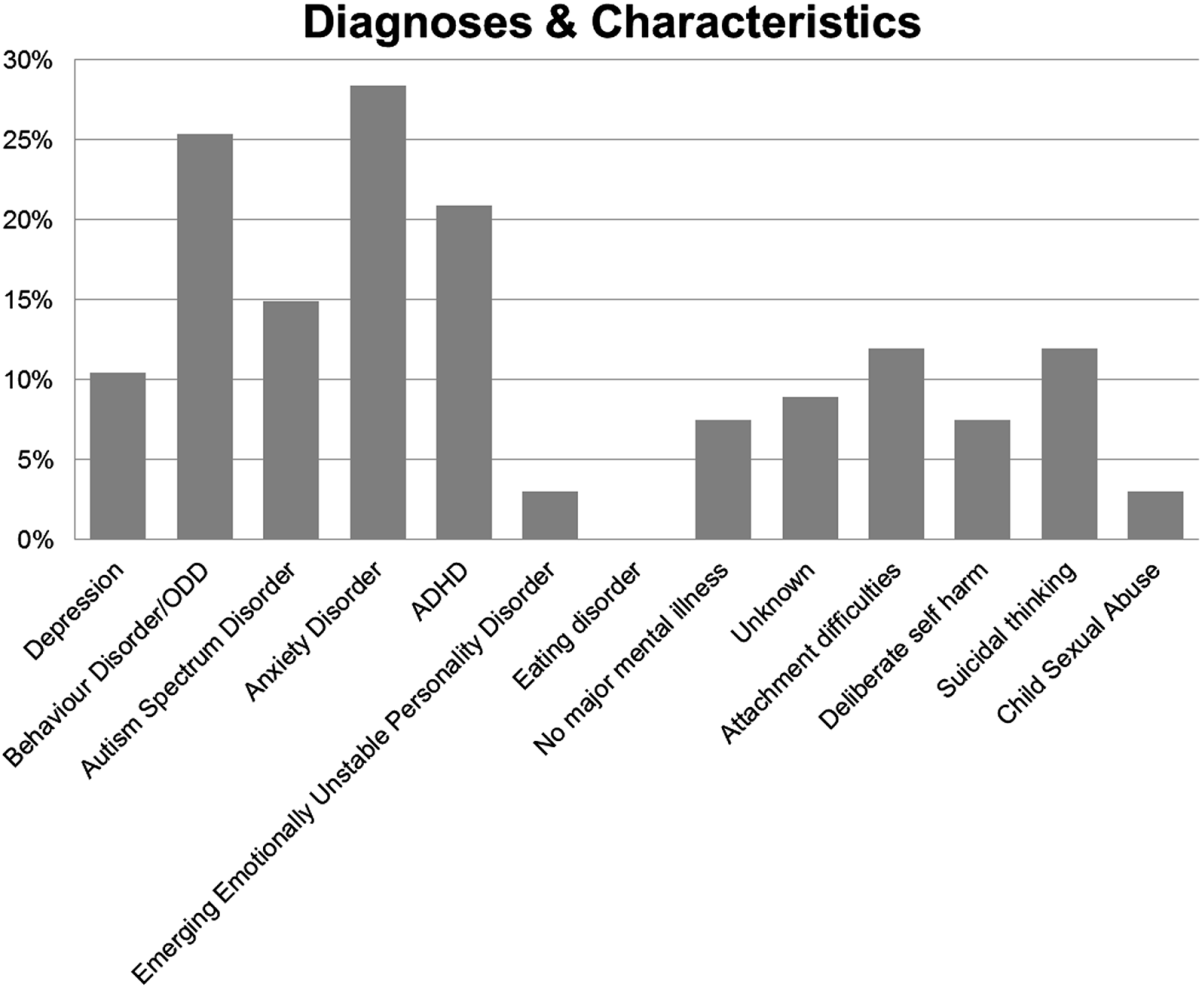
Diagnoses and characteristics of patients referred to CAP

The children referred to CAP showed a variety of clinical conditions as can be seen in Fig. 1.

We found that anxiety disorder (28 %), behaviour disorder/ODD (25 %), and ADHD (21 %) are the most common diagnoses referred to CAP. Children with attachment difficulties (12 %) and those who experienced suicidal thinking (12 %) are also frequently referred (Fig. 1). No children with eating disorders were referred to CAP and just 2 children with a history of CSA were referred to CAP. 7.4 % of those referred to CAP (N = 5) had experienced an Acute Life Event, 45 % were considered to have familial relationship issues or communication difficulties in the home; 3 % had stressors outside the home and 24 % had no significant distortion of the psychosocial environment. 6 (9 %) of children referred to CAP were in out of home placement; 40 lived with one or both parents and the status of 20 children was not recorded.

### Engagement in therapy

The 67 patients in the study attended a mean of 77.4 % (±20.1 %) of all appointments offered. There were significantly more appointments offered to males than to females (mean of 16.68 ± 14.6 vs 10.03 ± 9.1, t = −2.3, df = 62.8, sig < 0.05) and there were more males in the sample (N = 38) than females (N = 29). There was no significant difference found in the percentage of appointments attended by males in comparison to females.

23 (34 %) children engaged well, 20 (30 %) children partially engaged and 24 (36 %) did not engage well with the therapy. Children between the ages of 8–11 years were commonly referred to CAP. Children between the ages of 15–16 years were not commonly referred; however, the data suggests they engaged well when referred. However when the percentage attendance in the age groups 5–<10, 10–<14 and 14–18 years were compared using ANOVA, no statistically significant effect of age-group on attendance was found.

### Engagement and diagnosis and characteristics

**Fig. 2:**
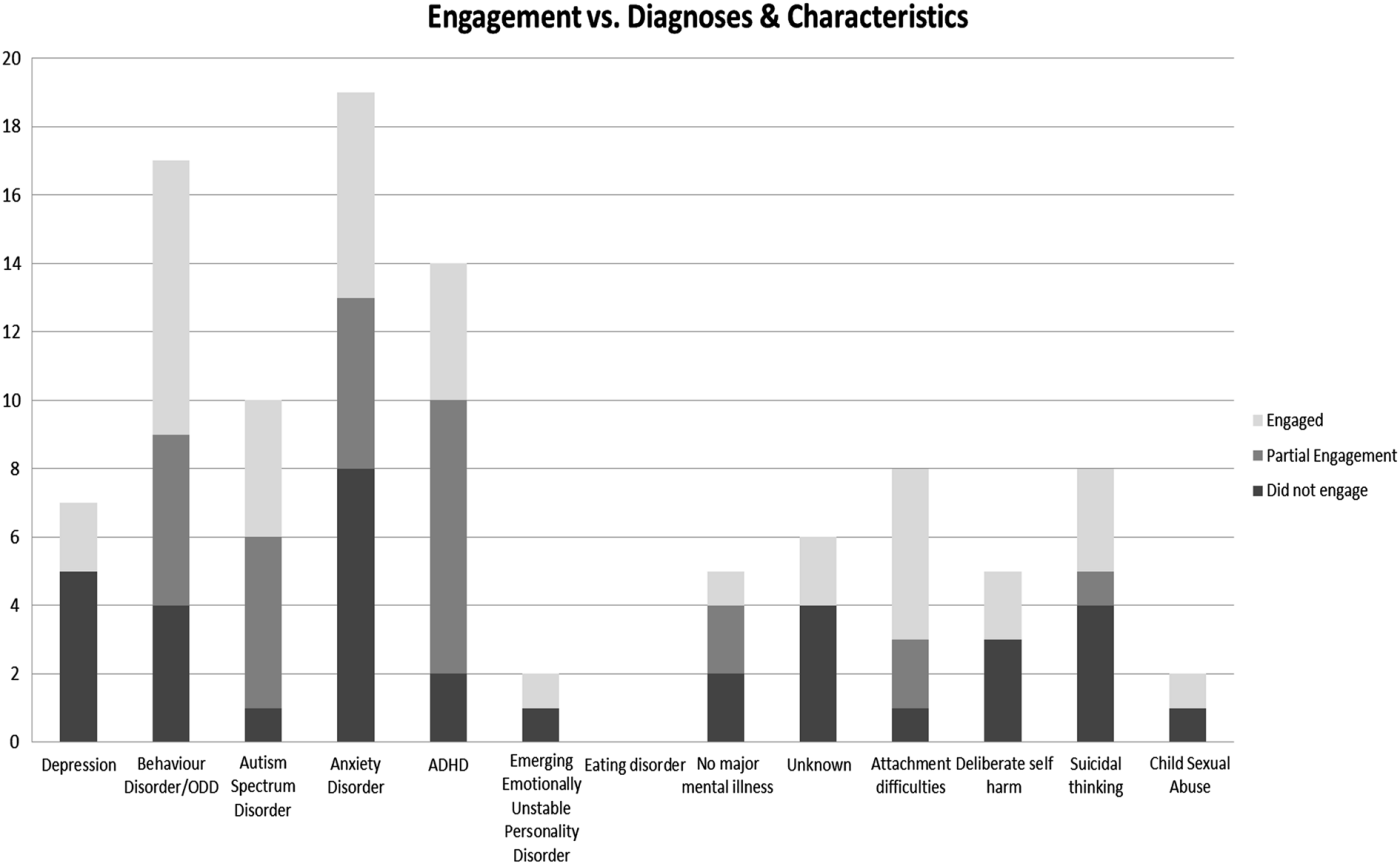
Relationship between engagement of children with CAP and their diagnostic categories and clinical characteristics

The engagement of children with each of the diagnostic categories can be seen in Fig. 2. Children with ADHD attended a mean of 23 sessions; children with anxiety disorder attended a mean of 11 sessions; children with ASD attended a mean of 19 sessions, children with behaviour disorder attended a mean of 13 sessions, children with depression attended a mean of 9 sessions, and children with no major mental illness attended a mean of 8 sessions. Children who were in out of home placement attended on average 11.3 sessions, which was not statistically significant from the average number attended by those who lived with a parent or parents (14.7 sessions). The 2 children who were victims of CSA attended on average 9 sessions. No significant effect of diagnosis was found on the percentage of appointments attended, using one way analysis of variance.

60 % of those who have experienced acute life events and 40 % of children with familial relationship issues or communication difficulties in the home engaged well with therapy. 100 % of those with outside home difficulties/stressors and 50 % of those with no significant distortion of the psychosocial environment did not engage well in CAP. Of the 6 children in out of home placement, 3 (50 %) of these engaged well with CAP and 2 partially engaged with CAP. 28 % (N = 11) of children who lived with a parent or parents engaged; 35 % (N = 14) had partial engagement and 37.5 % (N = 15) did not engage well. No statistically significant effect of these clinical characteristics was found on the percentage of appointments attended, using one way analysis of variance.

When engagement was compared in those with and without learning difficulties, we noted no statistically significant effect of learning difficulties was found on the percentage of appointments attended, using one way analysis of variance. As there were 16 therapists for 67 children we did not analyse engagement according to therapist.

## Discussion

We found that patients attended 77 % of CAP appointments offered in the clinic over the 13 year timescale of the study. It is interesting to note that this figure is similar to the attendance rate for first appointments in the same CAMHS in 2008, as a previous study found that 22.8 % of patients did not attend the first offered appointment (Sodipo et al. 2008). The attendance rate at CAP during the timescale of the study was higher than the overall attendance of 69 % in the same service in 2011 (FIOS Committee 2011) and similar to attendance rates today (80 %). This suggests that over a number of years CAP may have had higher attendance rates than treatment as usual in our clinic. The previous study in the service indicated a high attrition rate, and found that only 54 % of patients who attended their first appointment attended for the second appointments (Sodipo et al. 2008). We believe an attendance rate of 77 % in CAP compares favourably with this general clinic attendance.

It can be difficult to get children and adolescents to engage in psychotherapy (Oetzel and Scherer 2003; Kazdin 1996). Furthermore, it should be acknowledged that a child’s perceived difficulties may be different to their medical diagnosis—for example a child with ASD may find CAP useful to help with anxiety symptoms though the CAP may not change the child’s symptoms of ASD.

The 67 children from CAMHS attended an average of 14 sessions of CAP. We note that children aged 8–12 years were more commonly referred than children younger than 8 years or older than 12 years (Fig. 2). However, for those who were referred to CAP at 15 and 16 years of age, 50 and 75 % respectively engaged well with therapy. This, however, could be a chance finding due to small numbers referred. We also found that males were regularly referred to CAP, and that they attended for significantly longer periods than females. As CAP has no recommended duration of treatment, we calculated the child’s engagement in therapy can be estimated using the percentage of sessions attended, as well as overall number of sessions attended.

Attendance and engagement in therapy may be influenced by parental as well as by child and therapist factors. The CAMHS clinic studied is in an urban area of high social need, and provides services to areas of extreme poverty and high crime. We expect that engagement in CAP may be higher if other family factors were maximised, such as the prevision of childcare facilities for younger siblings, which may improve the parent’s ability to bring a child who needs therapy to regular appointments. Our data suggested that children who lived in “out of home placement” engaged better than those who lived at home, though this finding was not statistically significant. However it is possible that looked after children may have better attendance as they are brought to the clinic by their carers whose funded role it is to organise attendance. Children in “out of home placement” may also present with more psychological distress and trauma than other children, and may then engage well with psychotherapy as they find it a useful space to process their traumatic experiences.

There is considerable evidence for the effectiveness of psychotherapy in children with various mental health disorders (Carr and Irish Council for Psychotherapy 2007). Randomised controlled trials have shown that art psychotherapy is effective in the treatment of aggression in children (Hashemian and Jarahi 2014; Alavinezhad et al. 2014), as an adjunctive treatment in the management of cancer (Monti et al. 2006; Svensk et al. 2009), and in the treatment of asthma in children (Beebe et al. 2010). The effectiveness of formal psychotherapy with planned endings in adults with mental health disorders has been shown in a large study of 26,000 adults and adolescents in the UK, and it has been shown that the duration or type of psychotherapy offered did not determine effectiveness (Stiles et al. 2015). Accessing formal psychotherapy for children similar to that described by Stiles et al. (2015) can be difficult in the Irish healthcare setting, especially considering that psychotherapists are not listed as part of the multidisciplinary CAMHS team (Health Service Executive 2006). It has been shown that formal psychotherapy is difficult to access in CAMHS clinics in Ireland (Mulligan 2016), whereas in the UK it is recommended that psychotherapists and creative therapists are part of the CAMHS teams (Joint Commissioning Panel for Mental Health 2013).

We found that anxiety disorder, behaviour disorder/ODD, as well as ADHD were the most common diagnoses referred to CAP. Children with attachment difficulties and those who experienced suicidal thinking were also frequently referred. We note that no child was referred to CAP with psychosis or with an eating disorder. We expect that these serious and rare conditions are referred to other therapeutic modalities in CAMHS. However, we note that NICE guidelines recommend Art Therapy as an adjunctive therapy in young people with psychosis (National Collaborating Centre for Mental Health 2014).

It was noted that children with ADHD attended more than the average number of sessions, attending for almost half a year on average, which is a considerably longer block of therapy than for most other disorders. Children with ADHD had a high rate of partial engagement (57 %) and a low rate of poor engagement (14 %), which compares with a poor engagement rate of 36 % in all children in the study. Considering that children with ADHD can be impulsive and impatient we consider it a very positive finding that they engaged so well in CAP. Their high rate of partial engagement may reflect disorganisation in their homes, which may be related to intergenerational ADHD or parent factors (Winnicott 1960). We suggest that CAMHS teams should refer children with behaviour disorders or ADHD to CAP as an adjunctive treatment to other therapies, especially considering that it has been shown that art therapy may reduce aggressive tendencies in children (Hashemian and Jarahi 2014; Alavinezhad et al. 2014).

We note that traditional teaching on the Vasarhelyi method of CAP has been that children with ASD should not be referred to CAP as they may not have the ability to reflect on their image making. However children with ASD in our study engaged well and attended for prolonged blocks of CAP, with an average of 19 sessions attended. The high engagement and their long attendance suggests that children with ASD find CAP useful, though it does not indicated effectiveness and the authors do not expect that CAP can change the autistic disorder. It should be noted that children attending CAMHS who have a diagnosis of ASD may have milder autistic symptoms than children attending a specialist autism service, who may be non-verbal. There is considerable interest in the literature in the provision of art therapy to children with ASD (Martin 2009) and there is some limited evidence to indicate that a family based art therapy programme may be effective in ASD (Moghaddam et al. 2016). It has been suggested that as the therapist adapts to the child’s interests, art therapy can provide a safe environment for these children and can act as a communicative tool to encourage both verbal and non-verbal expression (Schweizer et al. 2014).

We note that just 29 % of patients with depression engaged well with CAP in CAMHS and hence we suggest that children with depression should be referred to another therapeutic modality in CAMHS. It is possible that the children simply did not have the motivation to attend a weekly service, and may have had a high drop-out rate from any therapy offered. There are other therapeutic modalities of proven benefit in the treatment of depression including cognitive behavior therapy and selective serotonin reuptake inhibitors (Goodyer et al. 2008; March et al. 2007), though we note current recommendations that “a specific psychological therapy (individual CBT, interpersonal therapy, family therapy, or psychodynamic psychotherapy)” is offered for at least 3 months in moderate to severe depression prior to commencing a selective serotonin reuptake inhibitor (National Institute for Health and Care Excellence 2005).

Therapist factors are generally considered to be important in engagement with therapy and the success of therapy (Roos and Werbart 2013). However, as there were 16 therapists who treated 67 children, the number treated by each therapist was small and we did not analyse engagement according to therapist.

There are a few limitations with this study. First, the sample size is modest and only based on data from 2 CAMHS teams. However as the data was collected over a number of years over which there were a number of changes in consultant and other staff on the CAMHS teams, we believe that the data reflects the referral practices of a relatively large group of CAMHS professionals. It is a limitation that we did not collect and analyse data on the ten children who were referred to CAP but did not attend any CAP sessions—this may have been due to a change in the team decision on how to treat the child or due to lack of interest in the child or parent in the therapy. It is also a possible limitation that we analysed data according to the main diagnosis indicated on the clinical records (e.g. ADHD), rather than according to the actual reason the child was referred to CAP (e.g. upset by recurrent school suspensions). However, this gives us data of interest in our day-to-day work in CAMHS. It is also a limitation that this is a retrospective chart review. More detailed prospective studies are underway, but will take some time to come to publication.

It is a strength of our study that a detailed database of clinical records with ICD categories which were assigned by the treating CAMHS clinician at the time the child attended CAMHS were available. This is valuable objective information. It is also a strength that the CAP attended by the children was provided by a therapist attending an MSc training programme, and hence it can be expected that the standard and method of CAP delivered to each of the children was comparable.

## Conclusion

We have shown that CAP is generally acceptable to children, with a high average attendance rate. Some children attended for more than a year of regular CAP sessions. It was noted that children with ADHD and with ASD engaged well with the therapy for prolonged periods, whereas children with depression did not engage so well. We suggest that CAMHS clinics should consider referring children diagnosed with ADHD and children diagnosed with ASD to CAP as an adjunct to other therapies. We suggest that individuals with depression should be referred initially to other therapeutic services as the engagement with CAP was relatively poor.
